# Peripheral Arterial Thrombosis following Russell's Viper Bites

**DOI:** 10.1055/s-0043-1769625

**Published:** 2023-06-17

**Authors:** Subramanian Senthilkumaran, Ketan Patel, Elanchezhian Rajan, Pradeep Vijayakumar, Stephen W. Miller, Alexandra Rucavado, Soheil Gilabadi, Medha Sonavane, Nicholas J. Richards, Jarred Williams, Harry F. Williams, Steven A. Trim, Ponniah Thirumalaikolundusubramanian, José María Gutiérrez, Sakthivel Vaiyapuri

**Affiliations:** 1 Department of Emergency Medicine, Manian Medical Centre, Erode, Tamil Nadu, India; 2 School of Biological Sciences, University of Reading, Reading, United Kingdom; 3 School of Pharmacy, University of Reading, Reading, United Kingdom; 4 The Poison Control Center, Children's Hospital of Philadelphia, Philadelphia, Pennsylvania, United States; 5 Instituto Clodomiro Picado, Facultad de Microbiología, Universidad de Costa Rica, San José, Costa Rica; 6 Toxiven Biotech Private Limited, Coimbatore, Tamil Nadu, India; 7 Venomtech Limited, Sandwich, United Kingdom; 8 The Tamil Nadu Dr M.G.R. Medical University, Chennai, Tamil Nadu, India

**Keywords:** Russell's viper, snakebite envenomation, thrombosis, peripheral arteries, venom

## Abstract

Envenomings by Russell's viper ( *Daboia russelii* ), a species of high medical importance in India and other Asian countries, commonly result in hemorrhage, coagulopathies, necrosis, and acute kidney injury. Although bleeding complications are frequently reported following viper envenomings, thrombotic events occur rarely (reported only in coronary and carotid arteries) with serious consequences. For the first time, we report three serious cases of peripheral arterial thrombosis following Russell's viper bites and their diagnostic, clinical management, and mechanistic insights. These patients developed occlusive thrombi in their peripheral arteries and symptoms despite antivenom treatment. In addition to clinical features, computed tomography angiography was used to diagnose arterial thrombosis and ascertain its precise locations. They were treated using thrombectomy or amputation in one case that presented with gangrenous digits. Mechanistic insights into the pathology through investigations revealed the procoagulant actions of Russell's viper venom in standard clotting tests as well as in rotational thromboelastometry analysis. Notably, Russell's viper venom inhibited agonist-induced platelet activation. The procoagulant effects of Russell's viper venom were inhibited by a matrix metalloprotease inhibitor, marimastat, although a phospholipase A _2_ inhibitor (varespladib) did not show any inhibitory effects. Russell's viper venom induced pulmonary thrombosis when injected intravenously in mice and thrombi in the microvasculature and affected skeletal muscle when administered locally. These data emphasize the significance of peripheral arterial thrombosis in snakebite victims and provide awareness, mechanisms, and robust strategies for clinicians to tackle this issue in patients.

## Introduction

Snakebite envenoming (SBE) has been classified as a high-priority neglected tropical disease by the World Health Organization. SBE results in around 140,000 deaths and 500,000 permanent disabilities annually worldwide. 1 2 Around 58,000 deaths occur every year in India alone, resulting in it being regarded as the “snakebite capital of the world.” 3 Russell's viper ( *Daboia russelii* ) is one of the “big four” venomous snakes (together with the Indian cobra [ *Naja naja* ], common krait [ *Bungarus caeruleus* ], and saw-scaled viper [ *Echis carinatus* ]) in India and is responsible for a majority of venomous bites resulting in deaths and disabilities. 4 5 6 7 The consequences of Russell's viper bites typically range from mild local effects to severe local necrosis and muscle damage as well as systemic effects such as hemorrhage, coagulopathies, neurotoxicity, and acute kidney injury. 8 Most SBE victims reside in rural, impoverished areas that lack adequate resources to handle these medical emergencies. 9 Moreover, victims who lack essential awareness of SBE often seek traditional treatments and other unproven methods thus delaying proven, time-sensitive treatments. 6 7 Delay in seeking appropriate treatment exacerbates envenoming effects resulting in serious consequences including death or loss of limbs. 7

Snake venoms contain a range of toxins which can affect multiple organs, tissues, and the blood coagulation system. 10 11 Due to the nature of toxins present in its venom, Russell's viper bites may lead to transient, asymptomatic coagulopathies or overt hemorrhage in the bite site as well as in internal organs including the kidneys, brain, lungs, and spleen, often with poor prognosis. 12 13 14 Russell's viper bite–induced bleeding complications are commonly observed, and they often lead to fatalities or permanent disabilities. 14 15 16 Similar to many other viper venoms, 12 13 Russell's viper venom induces consumption coagulopathy which results in rapid depletion of coagulation factors owing to the action of procoagulant enzymes, and this contributes to excessive bleeding. 14 17 Venom procoagulant activities may also result in thrombosis in the microvasculature and less commonly in large blood vessels which can lead to ischemic stroke. 18 19 20 SBE-induced ischemic stroke has been reported to cause bilateral blindness with permanent disabilities in rare cases. 21 Sudden cardiac arrest following SBE including Russell's viper envenoming has also been reported. 22 23 While bleeding complications are often reported for Russell's viper as well as other viper bites, peripheral arterial thrombosis (specifically in limbs) following SBE has never been reported. Even the envenomings by the Caribbean endemic species, *Bothrops lanceolatus* , which often induce pulmonary embolism, myocardial infarction, and cerebral ischemia did not cause peripheral arterial thrombosis in limbs. 24 25 Here, we report three serious cases of peripheral arterial thrombosis in upper limbs following Russell's viper bite envenomings and discuss their underlying molecular mechanisms and clinical management strategies. This study provides essential awareness of the life-threatening impacts of SBE, which is urgently needed and adds to our growing understanding of the pathophysiology of envenomings by Russell's viper.

## Results

### Clinical Presentation of Patients

**Fig. 1:**
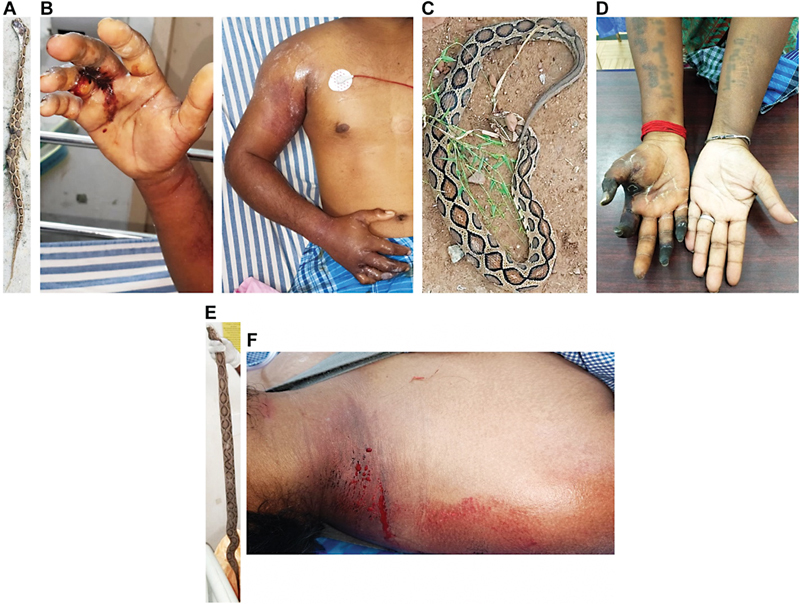
The offending Russell's viper specimens and local envenoming effects in victims. ( **A** ) The offending snake species of the first patient was identified as Russell's viper by a herpetologist. ( **B** ) Russell's viper bite induced bleeding at the bite site and swelling and discoloration of the right arm of the first patient. ( **C** ) The offending snake species which was confirmed as Russell's viper for the second patient ( **D** ), who displayed gangrenous digits (including the index finger where the bite occurred) in their right hand. The specimen of Russell's viper ( **E** ), which bit the third patient at the nape of the neck and caused local bleeding ( **F** ).

The first patient was a 21-year-old male auto-rickshaw driver who was bitten by a snake on a finger in his right hand while cleaning his vehicle near a pond in Sathyamangalan within the Erode district of Tamil Nadu (South India). The offending snake was identified as Russell's viper by an experienced herpetologist ( Fig. 1A ). The patient presented at a local hospital 75 minutes after the bite with symptoms of fever, vomiting, pain, bleeding at the bite site, and swelling of the right upper arm. He was treated with 18 vials (i.e., 180 mL) of equine polyvalent antivenom (Biological E Limited, India) raised against the Indian “big four” venomous snakes 100 minutes after the bite occurred. There was no history of oliguria, hematuria, ptosis, neck or limb weakness, or myalgia. The initial diffuse pain became localized to the fingers of his right hand and increased in intensity. He was subsequently referred to our emergency department (Manian Medical Centre, Erode, Tamil Nadu, India) 22 hours after the bite for further management. On examination, there was severe tenderness up to the axilla and limb swelling ( Fig. 1B ). The entire limb was discolored, cold and clammy, and movement of the affected limb and fingers caused severe pain. Transthoracic echocardiography showed an ejection fraction of 70%, an absence of valvular pathology, and normal systolic and diastolic function. Laboratory investigations indicated normal values of D-dimer, fibrinogen, hemoglobin, platelet count, prothrombin time (PT), and activated partial thromboplastin time (aPTT), along with other parameters ( Table 1 ). His chest and neck X-ray as well as a chest computed tomography (CT) scan failed to reveal any thoracic outlet syndrome.

**Table 1:** Laboratory investigation results using clinical samples obtained from patients upon admission to our emergency department

Specimen	Investigation	Patient 1	Patient 2	Patient 3	Unit	Normal range
EDTA blood	Hemoglobin	13.2	11.3	13.8	g%	12.0–16.0
EDTA blood	Total RBC count	4.74	4.99	4.82	Millions/μL	4.00–5.50
EDTA blood	HCT	39.8	35.4	41.0	%	33.00–50.00
EDTA blood	MCV	84.0	70.9	85.1	fl	81.10–96.00
EDTA blood	MCH	27.8	22.6	28.6	pg	27.20–33.20
EDTA blood	MCHC	33.2	31.9	33.7	%	32–36
EDTA blood	Total WBC count	11.88	6.26	13.91	×10 ^3^ cells/µL	4.00–11.00
EDTA blood	Neutrophils	8.09	3.62	11.71	×10 ^3^ cells/µL	2.0–7.0
EDTA blood	Lymphocytes	2.34	2.1	0.98	×10 ^3^ cells/µL	1.0–3.0
EDTA blood	Monocytes	1.03	0.42	1.21	×10 ^3^ cells/µL	0.1–0.8
EDTA blood	Eosinophils	0.37	0.1	0.0	×10 ^3^ cells/µL	0.02–0.5
EDTA blood	Basophils	0.05	0.02	0.01	×10 ^3^ cells/µL	0.02–0.1
EDTA blood	Neutrophils	68.1	57.9	84.2	%	55–75
EDTA blood	Lymphocytes	19.7	33.5	7.0	%	15–30
EDTA blood	Eosinophils	3.1	1.6	0.0	%	1–5
EDTA blood	Monocytes	8.7	6.7	8.7	%	2–10
EDTA blood	Basophils	0.4	0.3	0.1	%	Up to 1
EDTA blood	Platelet count	240	336	319	×10 ^3^ cells/µL	150–450
EDTA blood	MPV	11.0	9.4	9.4	fl	6.5–12.0
EDTA blood	PDW	13.5	9.6	9.8	fl	9.0–13.0
Serum	Urea	21.4	14.98	14.98	mg/dL	15–40
Serum	Creatinine	1.02	0.76	0.89	mg/dL	0.6–1.4
Serum	Uric acid	4.7	5.0	6.9	mg/dL	3.4–7.2
Citrated plasma	Prothrombin time	13.8	14.3	26.2	Seconds	11.6 (control)
Citrated plasma	INR	1.26	1.31	2.39	Ratio	
Citrated plasma	aPTT	35.2	34.1	34.3	Seconds	26–40
Serum	Bilirubin (total)	0.85	0.68	1.0	mg/dL	0.2–1.2
Serum	Bilirubin (direct)	0.32	0.28	0.38	mg/dL	0–0.2
Serum	Bilirubin (indirect)	0.53	0.4	0.62	mg/dL	0.2–0.9
Serum	SGOT	16	14	35	U/L	5–35
Serum	SGPT	14	15	27	U/L	5.0–45

Abbreviations: aPTT, activated partial thromboplastin time; HCT, hematocrit; INR, international normalized ratio of clotting; MCH, mean corpuscular hemoglobin; MCHC, mean corpuscular hemoglobin concentration; MCV, mean corpuscular volume; MPV, mean platelet volume; PDW, platelet distribution width; RBC, red blood cells; SGOT, serum glutamic oxaloacetic transaminase; SGPT, serum glutamic pyruvate transaminase; WBC, white blood cells.

The second patient was a 35-year-old female housewife who was bitten on the right index finger by a snake while cleaning her cattle shed near Perundurai in the Erode district of Tamil Nadu. The snake was identified as Russell's viper ( Fig. 1C ) by a herpetologist. The patient had no history of underlying diseases such as cancer, pregnancy, oral contraceptives, or type 2 diabetes mellitus and was COVID-19 negative. No underlying conditions were observed that would result in increased levels of procoagulant zymogens, decreased levels of coagulation inhibitors, or fibrinolytic abnormalities. She presented to a local hospital 90 minutes after the bite with severe pain and bleeding at the bite site and was treated with 20 vials (i.e., 200 mL) of polyvalent antivenom (VINS Bioproducts Limited, India) around 2 hours after the bite and given intravenous administration of broad-spectrum antibiotics. During the next 48 hours, she developed sudden onset of pain and swelling of the right hand which extended up to the elbow and was accompanied by pain and discoloration of the thumb and index finger. These symptoms persisted and she was referred to our emergency department (Manian Medical Centre) 4 days after the bite as there was no improvement. Physical examination revealed absent pulses of her right radial artery and well-defined gangrene of the distal phalanges of the right thumb, ring finger, and little finger, as well as the entire index finger ( Fig. 1D ). The neurological assessment revealed a lack of nociception in the affected area. Patent foramen ovale was ruled out as a cause of paradoxical embolism with transthoracic echocardiography. Tests for lupus anticoagulant, factor V Leiden, and prothrombin allele mutations were all negative. The levels of antithrombin, proteins C and S, and all other parameters ( Table 1 ) were within the normal limits.

The third patient was a 32-year-old male farmer who, while picking fruits in his farmland near Gobichettipalayam (Erode district, Tamil Nadu) was bitten by a Russell's viper (as confirmed by a herpetologist; Fig. 1E ) on the nape of his neck ( Fig. 1F ). He was admitted to our emergency department (Manian Medical Centre) within 90 minutes of the bite with a painful and swollen right upper limb, oozing blood from the bite location, and bleeding gums. The patient had no history of underlying diseases/conditions such as cancer and type 2 diabetes mellitus and was negative for COVID-19. There were no other diagnosed conditions relating to clotting abnormalities. On examination, the limb was swollen up to the hand, clammy and discolored. A 20-minute whole blood clotting test (WBCT20) for coagulopathy was performed and found to be prolonged ( Table 1 ). Since the patient presented with both local and systemic signs and symptoms of envenoming, he was treated with 10 vials of (i.e., 100 mL) polyvalent antivenom (Bharat Serums and Vaccines Limited, India) within 2 hours after the bite. Hematologic, metabolic, biochemical, and coagulation parameters were found to be within normal limits after 4 hours of antivenom administration. Despite antivenom treatment, the patient developed a painful, swollen area in the right retro clavicular, supraclavicular region and anterior side of the right upper arm. He subsequently developed wrist drop, inability to flex all fingers, and total loss of sensation in the right arm. Both active flexion and passive extension of the elbow produced severe pain. The examination discovered an absent brachial, ulnar, and radial pulse. He was found to have 60% oxygen saturation in his right middle finger; however, all other right-hand fingers had 0% saturation and no flow tracing. There was no evidence of intracardiac thrombus or shunt on the transthoracic echocardiogram. The patient's thrombophilic profile (factor V Leiden, prothrombin mutation, homocysteine, and deficiencies for protein C, protein S, and antithrombin III) was unremarkable.

The antivenom in all patients was administered in line with the standard protocols (i.e., 10 vials stat over 30 minutes and then 6 vials every 6 hours based on the symptoms) provided by the Government of India for the management of snakebites ( https://nhm.gov.in/images/pdf/guidelines/nrhm-guidelines/stg/Snakebite_QRG.pdf ).

### CT Angiography Revealed the Presence of Thrombi in Peripheral Arteries

**Fig. 2:**
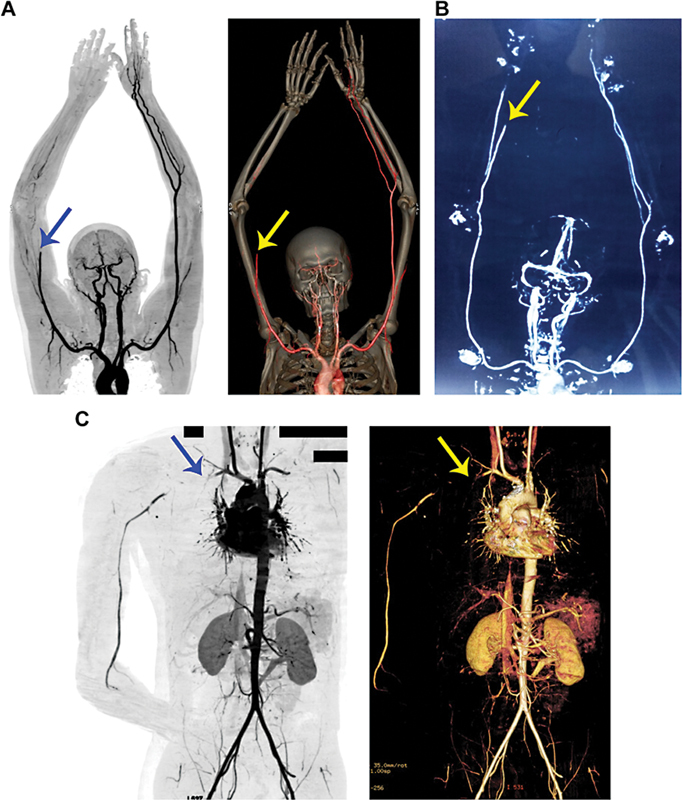
CT angiography reveals occluded peripheral arteries and a lack of downstream blood flow in Russell's bite victims. ( **A** ) The 2D contrasting and 3D constructed images of CT angiography confirm the occlusion of the right brachial artery and the lack of blood flow to downstream arteries in the first patient. ( **B** ) The 2D inverted contrasting image of CT angiography confirms the occlusion of the radial artery and blockade of blood flow downstream in the hand and fingers in the second patient. Similarly, 2D and 3D CT angiography ( **C** ) images confirm the occlusion of the right subclavian artery and the affected downstream blood flow in the third patient. The arrows indicate the site of occlusion. The black rectangle boxes are used to hide personal details on the image.

The clinical features such as pain, numbness, change in skin color, absence of pulses, and limited range of motion in these patients suggested that thrombosis might have occurred in their peripheral blood vessels. Compartment syndrome was suspected in the first patient and therefore a preliminary Doppler ultrasound was performed. This revealed impaired blood flow to the extreme forearm arteries indicating acute limb ischemia. Subsequently, he was evaluated with a peripheral CT angiogram which showed complete occlusion of the right brachial artery ( Fig. 2A ). Similarly, a peripheral CT angiogram in the second patient revealed distal radial artery occlusion and short-segment occlusion in the palmar arterial arch between the thumb and the index fingers ( Fig. 2B ). In addition, one of the two digital arteries of the thumb and both digital arteries of the index finger were occluded. For the third patient, a clinical diagnosis of compartment syndrome with vascular compromise was assumed and, therefore, an urgent CT angiogram was performed. This revealed occlusive thrombi in the right mid-subclavian artery and subclavian vein, axillary, and brachial veins ( Fig. 2C ). Arteries distal to the mid-subclavian artery showed minimal flow up to the wrist level. A large hematoma was seen in the right retro clavicular and supraclavicular region compressing the third part of the subclavian artery.

### Clinical Management of Thrombosis in Patients

Following the confirmation of thrombosis, an emergency thrombectomy was performed in the first patient and successfully removed a thrombus measuring around 4 inches. He was then treated with intravenous administration of low-molecular-weight heparin and oral warfarin. The patient also underwent two fasciotomies which reduced swelling and tenderness in the affected limb and improved movement. A Doppler ultrasound scan performed 3 days after the thrombectomy showed normal blood flow in the arteries of the affected limb. Upon discharge, he was prescribed warfarin for another 6 weeks and advised to follow up for regular monitoring of PT and the international normalized ratio (INR) of clotting. Since the second patient arrived with significant gangrenous tissues on the fingers, reperfusion therapy was not possible. Based on the occlusive thrombosis and resulting gangrene, the decision was made to amputate the patient's entire thumb, index, and ring fingers, as well as the little finger at the middle phalanges. The thrombus (around 3–4 inches in size) at the distal radial artery was removed by thrombectomy and the patient was started on anticoagulant therapy similar to the first patient. Unlike the other two patients, in the third patient, an emergency subclavian thrombectomy was performed via transbronchial route using a Fogarty catheter (due to the location of the thrombus) which successfully removed a thrombus with the size of around 4 inches and rescued proximal flow. The patient was started on oral warfarin therapy and follow-up visits revealed that pulses were detectable in all distal vessels and the fingers had returned to normal function. The monthly regular check-ups were normal in all three patients. Follow-up blood tests including clotting factors such as V and X were performed 4 weeks after the discharge for all patients and we did not find any clotting or other relevant abnormalities.

### Russell's Viper Venom Exerts Procoagulant Effects

**Fig. 3:**
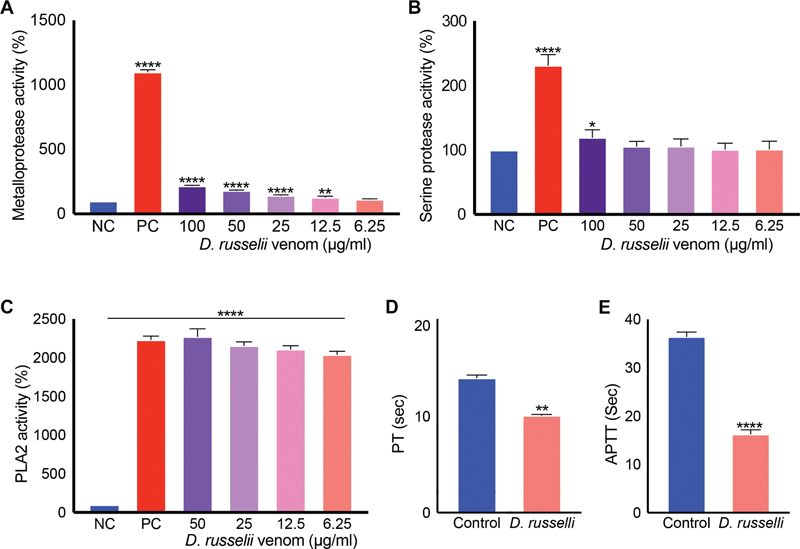
Enzymatic and clotting activities of Russell's viper venom. The metalloprotease ( **A** ), serine protease ( **B** ), and PLA _2_ ( **C** ) activities of various concentrations of Russell's viper venom were measured using respective fluorogenic substrates by spectrofluorimetry. The base level fluorescence obtained with negative controls (NC; i.e., the substrate in the absence of venom) at 90 minutes was taken as 100% to calculate the enzyme activities in venom samples at the same time point. The venom of *Crotalus atrox* (50 μg/mL) was used as a positive control (PC) in all these assays. 50 μg/mL Russell's viper venom was mixed with plasma and relevant reagents to measure PT ( **D** ) and aPTT ( **E** ) using Ceveron T100 fully automated coagulation analyzer. Data represent mean ± S.D. ( *n* = 4). The *p* -values (* *p* < 0.05, ** *p* < 0.01 and **** *p* < 0.0001) shown were calculated by one-way ANOVA ( **A** – **C** ) or unpaired *t* -test ( **D** and **E** ) using GraphPad Prism.

Since Russell's viper bites are frequently reported to cause bleeding complications associated with incoagulable blood due to venom-induced consumption coagulopathy, the development of occlusive thrombi in peripheral arteries is surprising. To determine the actions of Russell's viper venom (pooled venom from multiple specimens collected at the Kentucky Reptiles Zoo, United States) on various coagulation parameters, in vitro coagulation experiments were performed. While Russell's viper venom displayed low metalloprotease ( Fig. 3A ) and serine protease ( Fig. 3B ) activities compared with the positive control (same concentration of the venom of *Crotalus atrox* ), it exhibited high phospholipase A _2_ (PLA _2_ ) activity ( Fig. 3C ). In addition, the venom (50 μg/mL) significantly reduced PT ( Fig. 3D ) and aPTT ( Fig. 3E ) compared with the controls in citrated human plasma as a reflection of its procoagulant activity.

**Fig. 4:**
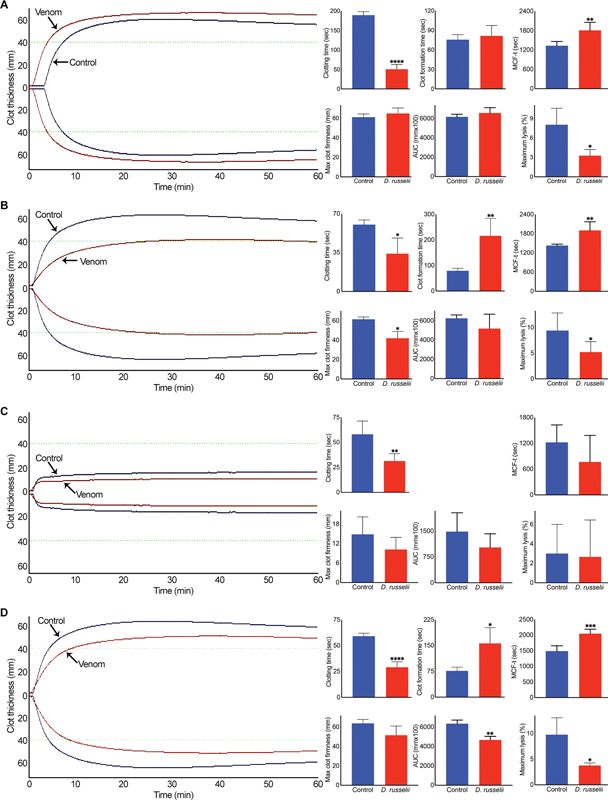
Effect of Russell's viper venom on ROTEM analysis. The impact of Russell's viper venom (50 μg/mL) on Intem ( **A** ), Extem ( **B** ), Fibtem ( **C** ), and Aptem ( **D** ) was analyzed by mixing the venom with citrated human whole blood and relevant reagents provided by the manufacturer and monitoring the clot formation over 60 minutes in a ROTEM Delta instrument. The curves shown are representative of four separate experiments performed using blood obtained from four individuals. Although various parameters were measured by ROTEM, here we demonstrate the impacts of venom on notable parameters such as clotting time (the time when clot formation was initiated), clot formation time (time to reach a 20-mm size clot), time to reach a maximum clot firmness (MCF-t), maximum clot firmness, area under the curve (AUC) of maximum clot formed, and maximum lysis. The cumulative data shown represent mean ± SD ( *n* = 4). The *p* -values (* *p* < 0.05, ** *p* < 0.01, *** *p* < 0.001, and **** *p* < 0.0001) shown were calculated by an unpaired *t* -test using GraphPad Prism.

To further examine the effects of Russell's viper venom on blood clotting, rotational thromboelastometry (ROTEM) analysis was performed using human-citrated whole blood. The Intem (which evaluates the intrinsic and common pathways) analysis confirmed the accelerated clotting when Russell's viper venom (50 μg/mL) was added to whole blood in comparison to the control as demonstrated through reduced clotting time and maximum clot lysis ( Fig. 4A ). However, there was no significant difference between the control and venom-induced clots in terms of size (area under the curve) and firmness. Similarly, the Extem (which evaluates the extrinsic and common pathways) analysis demonstrated accelerated clotting with reduced clot lysis, although the maximum size clot formation was significantly delayed ( Fig. 4B ). To determine the impact of Russell's viper venom on clotting independently from platelets, the Fibtem analysis was performed. This analysis also confirmed the accelerated clotting, although the firmness of the clot was reduced ( Fig. 4C ). Russell's viper venom also displayed accelerated blood clotting and reduced lysis when fibrinolysis was prevented using the Aptem analysis ( Fig. 4D ). Together, these data demonstrate that Russell's viper venom induces blood clotting through common (or both intrinsic and extrinsic) coagulation pathways independently from platelets and fibrinolysis.

### Russell's Viper Venom Inhibits Agonist-Induced Platelet Activation

**Fig. 5:**
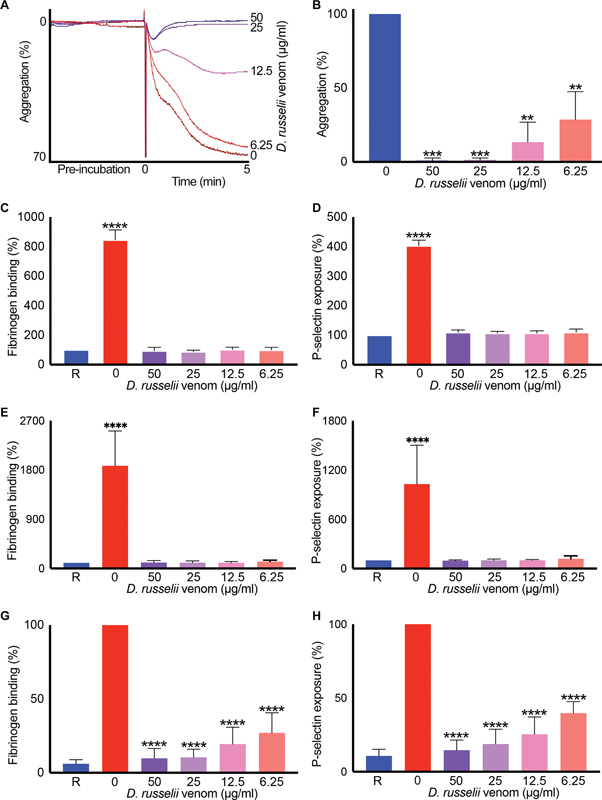
Impact of Russell's viper venom on human platelet activation. The human PRP was mixed with various concentrations of Russell's viper venom and incubated at 37 °C in an optical aggregometer while monitoring the level of aggregation for 5 minutes. Then the agonist, 5 μM ADP, was added, and the level of aggregation was monitored for another 5 minutes. The traces shown ( **A** ) are representative of four separate experiments. The level of aggregation obtained with the vehicle control (i.e., in the absence of venom) was taken as 100% to calculate the level of aggregation in venom-treated samples ( **B** ). The levels of fibrinogen binding and P-selectin exposure as markers for platelet activation were measured in the presence and absence of various concentrations of Russell's viper venom after 5 ( **C** and **D** ) and 20 ( **E** and **F** ) minutes of incubation without any platelet agonist. Similarly, the levels of fibrinogen binding ( **G** ) and P-selectin exposure ( **H** ) were measured following a 5-minute incubation with different concentrations of Russell's viper venom followed by 20-minute incubation with 5 μM ADP at 37 °C. The base level fluorescence obtained with relevant controls was taken as 100% to calculate the impact of venom in treated samples. Data represent mean ± SD ( *n* = 4). The *p* -values (** *p* < 0.01, *** *p* < 0.0001, and **** *p* < 0.0001) shown were calculated by one-way ANOVA using GraphPad Prism. “R” represents the level of activation in resting platelets.

Since Russell's viper venom induced blood clotting even in the absence of platelets, its direct effects on human platelets were studied. Different concentrations of Russell's viper venom failed to induce platelet aggregation on their own in human platelet-rich plasma (PRP), although they largely inhibited agonist (ADP)-induced platelet aggregation ( Fig. 5A, B ). To corroborate these effects, the levels of fibrinogen binding (as a marker for inside-out signaling to integrin αIIbβ3) and P-selectin exposure (as a marker for α-granule secretion) were quantified in the presence and absence of various concentrations of Russell's viper venom. Similar to the results obtained with aggregation, the various concentrations of venom failed to induce platelet activation on their own either following incubation of 5 ( Fig. 5C, D ) or 20 ( Fig. 5E, F ) minutes. However, they largely inhibited ADP-induced fibrinogen binding ( Fig. 5G ) and P-selectin exposure ( Fig. 5H ) on human platelets. Taken together, these data demonstrate that Russell's viper venom-induced clotting in vitro, an activity known to be responsible for consumption coagulopathy in vivo, occurs mainly via activation of coagulation factors/cascades. Furthermore, the inhibition of platelet function may contribute to bleeding complications together with consumption coagulopathy.

### Marimastat Inhibits the Procoagulant Activity of Russell's Viper Venom

**Fig. 6:**
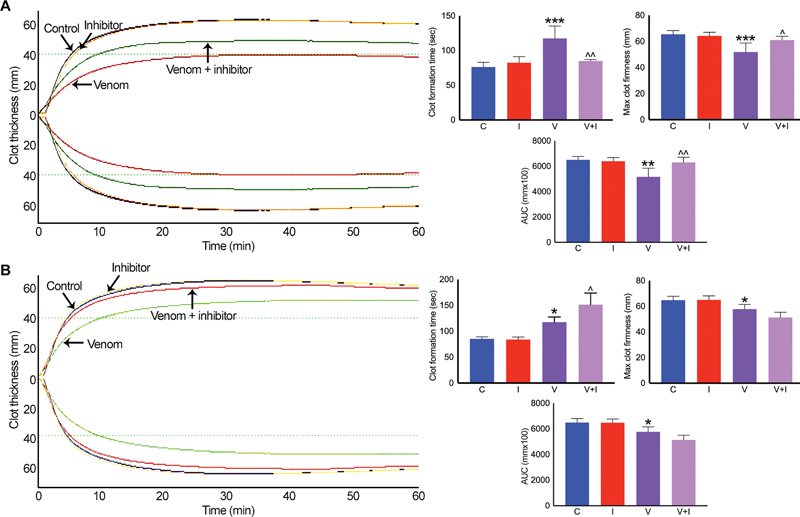
Effects of marimastat and varespladib on Russell's viper venom-induced clotting in ROTEM. 10 μM marimastat ( **A** ) or varespladib ( **B** ) was mixed with 50 μg/mL Russell's viper venom in citrated whole human blood before the addition of Extem reagents and monitoring the level of clot formation over 60 minutes in ROTEM. The traces shown are representative of four separate experiments performed using blood obtained from four donors. The cumulative data are shown for specific parameters such as clot formation time, maximum clot firmness, and area under the curve (AUC) for full clot formed. Data represent mean ± SD ( *n* = 4). The *p* -values (* *p* < 0.05, ** *p* < 0.01 and *** *p* < 0.0001) shown were calculated by one-way ANOVA using GraphPad Prism. C—vehicle control; I—inhibitor alone; V—venom alone; V + I—venom + inhibitor. ^*^ Significance when venom-treated samples compared with the controls. ^^^ Significance when venom and inhibitor-treated samples compared with the venom-alone controls.

A matrix metalloprotease inhibitor, marimastat, has been demonstrated to possess broad-spectrum inhibitory effects on venom metalloproteases. 26 27 Hence, to determine its effects on Russell's viper venom-induced procoagulant effects, 10-μM marimastat was incubated with 50 μg/mL venom for 5 minutes before mixing with human citrated whole blood and analyzing (Extem) in ROTEM. As shown earlier, the venom has reduced clotting time, firmness, and clot size, while it increased clot formation time for a 20-mm clot in Extem analysis compared with the controls ( Fig. 6A ). Although marimastat did not affect any parameters of Extem on its own, it inhibited the procoagulant actions of Russell's viper venom. Similarly, varespladib is a PLA _2_ inhibitor which has been shown to have significant impacts on venom PLA _2_ of numerous snake species. To determine if PLA _2_ in Russell's viper venom is involved in inducing clotting effects, 10-μM varespladib was mixed with 50 μg/mL venom before analyzing in Extem analysis. Varespladib on its own and with venom did not show any effects on Extem clotting parameters except a slight increase in clot formation time when mixed with the venom ( Fig. 6B ). These data demonstrate that the procoagulant effects of Russell's viper venom are largely mediated through metalloproteases instead of PLA _2_ , although we cannot rule out the possible contributions from other venom proteins.

### Histological Assessment of Thrombosis under In Vivo Settings in Mice

**Fig. 7:**
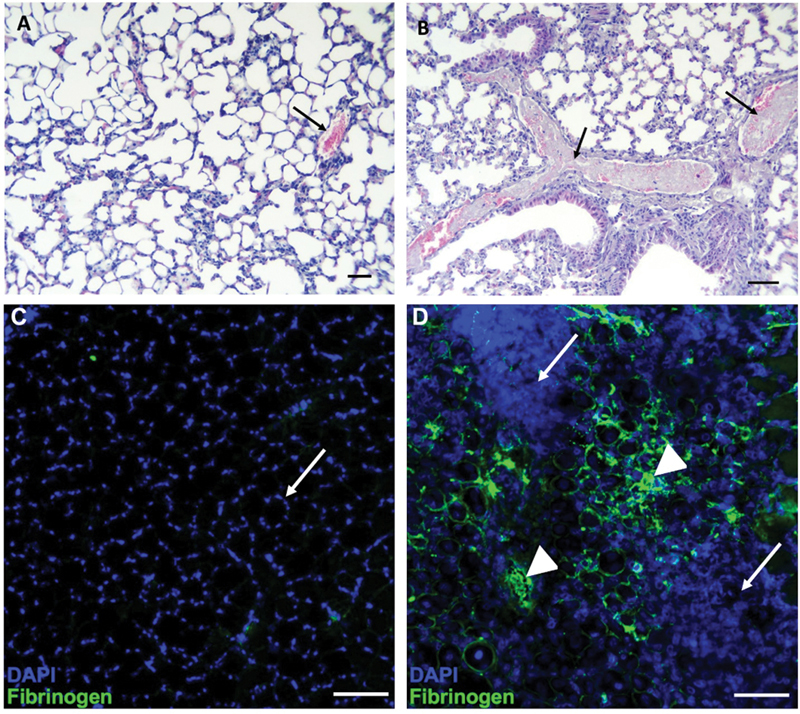
Russell's viper venom-induced thrombosis in mice. Light micrographs of lung tissues (stained with hematoxylin and eosin stain) of mice injected intravenously in the caudal vein, with PBS ( **A** ) or 12.5 µg Russell's viper venom previously incubated with the PLA _2_ inhibitor, varespladib ( **B** ) are shown. The tissue from control mice shows a normal histological pattern and a vein filled with erythrocytes (indicated by an arrow). In contrast, tissue from mice injected with Russell's viper venom shows two veins with prominent thrombi (indicated by arrows). Similarly, immunofluorescence images of the tibialis anterior muscle of mice injected with PBS or Russell's viper venom 5 days before dissection were obtained following staining with FITC-labeled anti-fibrinogen antibodies. ( **C** ) A section of the tibialis anterior muscle of control mice shows normal muscle architecture with nuclei at the periphery of muscle fibers (indicated by an arrow) and the lack of fibrinogen binding (no green signal). ( **D** ) The tibialis anterior muscle injected with Russell's viper venom (66 ng/g of mouse weight) shows extensive tissue damage with the destruction of muscle fibers as indicated by the presence of a large number of cells (arrow). Significant levels of fibrinogen found in the damaged regions (green color) demonstrate the presence of bleeding and thrombi (arrowheads). The bar represents 100 µm.

To determine if Russell's viper venom can acutely induce thrombosis in the vasculature, lung tissues obtained from mice injected intravenously with vehicle control (phosphate-buffered saline [PBS]) or Russell's viper venom were analyzed using hematoxylin and eosin staining. The tissues from control mice showed a normal histological appearance, and blood vessels were filled with erythrocytes without any thrombi ( Fig. 7A ). In contrast, mice that received Russell's viper venom inhibited with varespladib, to block the action of neurotoxic PLA _2_ s, died within 15 minutes of injection. Tissues from these mice showed abundant thrombi in pulmonary veins and venules ( Fig. 7B ). Noteworthy, edema, hemorrhage, and inflammatory infiltrate were absent in the lung tissue of envenomed mice. To further establish if Russell's viper venom induces thrombi in microvasculature and tissues, skeletal muscle (tibialis anterior) injected with vehicle control or Russell's viper venom was analyzed by immunohistochemistry using FITC-labeled anti-fibrinogen antibodies. The muscle sections from control mice showed normal tissue architecture 5 days after the injection. The nuclei were spaced at the periphery of muscle fibers giving the typical hollow fiber appearance. Notably, there was no binding of anti-fibrinogen antibodies to the control muscle sections ( Fig. 7C ). However, muscle injected with Russell's viper venom showed considerable disruption to tissue architecture evidenced by a massive invasion of cells leading to large areas covered by DAPI-positive nuclei, and a significant amount of fibrinogen deposition including large thrombi ( Fig. 7D ). These data demonstrate that Russell's viper venom can induce thrombosis in the vasculature in affected tissues.

## Discussion

Bleeding complications (hemorrhage) from SBE inflicted by many viperid species are commonly reported and they can lead to cerebral hemorrhage, 28 29 pituitary failure, 30 pseudoaneurysm, 15 perinephric hematoma, 31 32 and acute kidney injury. 33 These complications can also contribute to the onset of cardiovascular shock and multiple organ failure. However, thrombosis, specifically peripheral arterial thrombosis, is a rare (but serious) phenomenon following SBE. There have been scarce reports of cases with thrombosis as a consequence of viperid snakebites. 34 35 36 An exception to this trend of the low frequency of thrombotic phenomena in SBE is the envenoming by *Bothrops lanceolatus* , an endemic species in the Caribbean Island of Martinique. A significant proportion of patients bitten by this species develop thrombotic complications associated with infarctions in the lungs, heart, and brain 24 37 ; however, no effects on limbs were reported in the literature. The mechanisms behind this effect were not established, although it has been proposed to be related to the actions of metalloproteases (which are abundant in this venom) on the vasculature. 38 39 Russell's viper is a foremost medically important venomous snake in India and other Asian countries. The bites from this species in India mostly lead to extensive bleeding, cardiovascular disturbances, muscle necrosis, acute kidney injury, neurotoxic effects, and multiple organ failure. These envenoming effects may result in permanent disabilities or death if not treated promptly. A few cases of cerebral thrombosis following envenomings by Russell's viper have been reported. 40 41 42 43 44 However, to the best of our knowledge, the peripheral arterial thrombosis resulting in loss of function of affected limbs has not been reported previously following Russell's viper bites. Hence, for the first time, we report three serious cases where the patients developed large (3–4 inches) occlusive thrombi in their peripheral arteries of upper limbs following Russell's viper bites despite antivenom treatment. While these patients were treated successfully using thrombectomy, one patient had to undergo amputation of their fingers. These thrombotic events are uncommon compared with the high prevalence of bleeding diatheses that accompany envenoming by Russell's vipers. Therefore, this condition needs significant scientific and medical attention to provide timely diagnosis and appropriate treatment for SBE victims, and thereby prevent permanent disabilities or deaths.

Russell's viper envenoming typically results in a pattern of local swelling and bleeding at the bite site, lymphadenopathy, hypotension, coagulopathy, and systemic bleeding, 45 although the severity of symptoms varies widely based on the amount of venom injected. Moreover, other complications such as acute kidney injury, systemic capillary leakage syndrome, vascular damage in the pituitary gland, and neurotoxicity also occur following Russell's viper bites. 46 In addition to clinical symptoms, simple coagulation tests such as a 20-minute whole blood clotting test, PT and aPTT, and the levels of fibrinogen and D-dimers are used to ascertain coagulopathy in SBE (including Russell's viper) victims. 46 Based on the severity of other complications, ultrasound, Doppler, CT, and magnetic resonance imaging (MRI) scans are utilized to establish bleeding and thrombotic conditions as well as organ damage/failure in Russell's viper and other SBE victims. 15 32 The patients described in this study were relatively young (aged 21–35 years) and had no significant previous medical conditions. However, they developed thrombi in their peripheral arteries and displayed symptoms at various time points following bites. The laboratory investigations demonstrated normal clotting and metabolic profile in all patients indicating that the clotting might have occurred much earlier than their arrival at the emergency department. All of them were treated with polyvalent antivenom raised against the Indian “big four” venomous snakes. While in one case (the third patient who was admitted directly to the emergency department), the time between the bite and first antivenom administration was around 2 hours, in others these details were not available as they received treatments elsewhere before the admittance in the emergency department. Interestingly, antivenom treatment did not prevent the development of thrombi in these victims, possibly due to the rapid/transient actions of venom components in the circulation or to the fact that thrombosis might be the result of endogenous processes that, once established, cannot be reverted by antivenom. Thrombi were formed in the bitten limb of two victims who had bites on the hand or finger while the third patient bitten in the neck developed regional thrombi. CT angiography played a critical role in the diagnosis of thrombosis in these patients, and it allowed the evaluation of precise locations of thrombi. The patients were successfully treated with thrombectomy, although the second patient who presented 4 days after envenoming developed gangrene and thus the affected fingers and thumb had to be amputated. All these patients received anticoagulant therapy which aided in the prognosis postthrombectomy. The prognosis of the first and third patients demonstrates that the earlier intervention is critical in thrombotic conditions to restore adequate flow to the affected areas and prevent the loss of limbs.

Russell's viper venom largely contains serine proteases, metalloproteases, and PLA _2_ s and all of these are known to interfere with the coagulation cascades, disrupt endothelial function, and cause tissue necrosis. Specifically, it comprises procoagulant factor V and X activators, 47 48 and prothrombin activating enzyme, 49 all of which induce blood clotting in vitro, while under in vivo settings, their action predominantly results in consumption coagulopathy with a rapid reduction in the concentrations of fibrinogen and other clotting factors, leading to bleeding. In some cases, thrombocytopenia as well as an increase in plasma fibrinolytic activity may occur, thus generating fibrin degradation products. 50 Our experimental observations using standard clotting tests and ROTEM corroborate the procoagulant activity of this venom. Such in vitro procoagulant activity is responsible for the consumption coagulopathy 51 that is usually observed in patients suffering envenomings by this species. The inhibitory effects displayed on agonist-induced platelet activation are likely to play a role in overall hemostatic disturbances and subsequent bleeding induced by this venom. In addition to direct hemostatic alterations, venom metalloproteases may promote hemorrhage by cleaving various components of the basement membrane such as type IV collagen in blood vessels, 52 which can be potentiated by coagulopathy. Indeed, the use of marimastat (a matrix metalloprotease inhibitor) in ROTEM experiments confirms the impact of Russell's viper venom metalloproteases in inducing procoagulant effects. It appears that the PLA _2_ s present in this venom may not contribute to the procoagulant effects, as varespladib did not reverse these actions. We cannot rule out the possible actions of other venom components in inducing blood clotting. Overall, the predominant hemotoxic actions of Russell's viper venom are associated with consumption coagulopathy, incoagulable nature of blood, and bleeding diathesis, although thrombosis was unusually observed in the patients reported in this study. In these sporadic cases, a portion of venom might have been injected directly into the blood vessels, thus causing the rapid formation of thrombi which can become immovable at junctions of arteries where continuous clotting can occur without embolization despite high arterial shear conditions. Indeed, our data demonstrate that the direct injection of Russell's viper venom in the caudal vein can induce the formation of large thrombi.

The continuous development of thrombi over several hours after envenoming resulted in delayed symptoms and loss of function of limbs until thrombi became occlusive. These patients might have experienced a high degree of endothelial damage secondary to the actions of venom metalloproteases in the vasculature, leading to an increase in exposed tissue factor, collagen, or von Willebrand factor with the resultant blood clotting. The experimental results presented in this study demonstrate that the clotting cascades played a major role independently from platelets, although this is unusual for arterial thrombosis. The activation of clotting factors and subsequent thrombosis with minimal inputs from platelets normally occurs under venous circulation often leading to pulmonary embolism. 53 Indeed, our experimental observations in mice indicate that prominent thrombi are observed in pulmonary veins within minutes of intravenous injection of the venom, thus suggesting that such venous thrombosis is the result of the rapid action of procoagulant enzymes in the venom in the circulation. Furthermore, our work shows that thrombi develop not only in the lungs in animal models but also in skeletal muscle (i.e., locally affected muscle around the bite/injection site). The skeletal muscle tissue has a very high capacity to regenerate, exemplified by the rapid formation of muscle fibers a few days after total muscle fiber necrosis induced by cardiotoxin. However, following Russell's viper venom–induced damage, there was considerable evidence for unresolved tissue necrosis as well as significant levels of fibrinogen deposition indicative of thrombi formation in the microvasculature and affected tissues. This is possibly due to the sustained action of venom components acting to continually damage the tissue as well as leading to thrombus formation. Russell's viper venom also induces prominent inflammatory responses, 54 and thus, the increase in a plethora of inflammatory mediators in the blood may also induce the acquisition of a proinflammatory and procoagulant phenotype in endothelial cells, thus favoring the formation of thrombi. Furthermore, the specific characteristics of individuals may affect the clinical features following SBE. It is possible that a certain subset of patients might possess increased levels of specific coagulation factors such as factors V and/or X which may create a hypercoagulable status during envenoming. Other endogenous factors such as undiagnosed atherosclerosis might also contribute to a condition that favors arterial thrombosis in some patients. It is unknown if preexisting atherosclerosis (in blood vessels other than the ones imaged) in these patients may have played a role, although atherosclerotic risk increases with advanced age 55 and all of these patients were younger than 40 years. Therefore, it is likely that the thrombosis described in this study is multifactorial in its origin.

The regional variations of Russell's viper venom composition 56 57 58 and their impacts on clinical manifestations of envenomings were previously reported. 46 59 Indeed, we reported various unusual complications of Russell's viper envenomings in South India that could be attributable to the variations in venom composition. Hence, these three patients might have been bitten by Russell's vipers that might have had unusual quantities of procoagulant venom toxins resulting in stable occlusive thrombosis. Here, snake-related genetic factors could have also played a role in the development of thrombosis. For example, genetic variations in subpopulations of species might have increased the proportion of procoagulant enzymes. It is well known that venom composition varies geographically in Russell's vipers even within India, resulting in significant variations in antivenom efficacy. 57 58 Indeed, different subspecies of Russell's viper were identified across South Asia. 60 Hence, further studies with additional cases describing thromboembolic events would be required to determine if a geographic relationship exists. Although difficult to assess in the field, the variable amount of venom delivered in a single bite might also play a role. Thus, a combination of patient-specific and snake-specific features may also contribute to the likelihood of the development of occlusive thrombi in some cases.

In conclusion, the procoagulant actions of Russell's viper venom mostly result in consumption coagulopathy leading to the incoagulable nature of blood, which contributes to the local and systemic bleeding characteristic of these envenomings. Conversely, as shown in this study, in some victims, occlusive thrombosis may occur which may impair blood flow downstream of the clot and cause ischemia resulting in pain, numbness, and, if left untreated, excessive tissue necrosis leading to permanent disabilities. Therefore, robust diagnostic and management strategies are required to handle Russell's viper bite victims. In addition to the usual coagulation tests, CT angiography is required for the robust diagnosis of thrombosis. It should be performed early when patients complain of relevant symptoms following bites. If thrombosis is confirmed, prompt thrombectomy with minimal surgery or percutaneous catheterization should be performed to remove the thrombi and rescue blood flow. Moreover, anticoagulant therapy is necessary to prevent subsequent thrombosis postthrombectomy. The patients should be monitored for excessive bleeding phenotype while receiving anticoagulant therapy. Clinicians in areas endemic to SBE including Russell's viper should be aware of such serious complications.

## Methods

### Patients' Data Collection

The collection of data from snakebite victims for this study was approved by the Institutional Ethics Committee at Toxiven Biotech Private Limited (ICMR-Toxiven Ethics 2019–001/002). Written informed consent was obtained from all patients. The patients were treated using standard protocols and specialized treatments as described in this article.

### CT Angiography

Helical CT angiography was performed using a fourth generation multidetector CT scanner (GE Optima CT660 128 Slice, UK) with a 0.6-second gantry rotation period. The angiography was achieved by acquiring four rows of 1.25-mm sections at a pitch of 1.5 following intravenous administration of 100 to 150 mL of iohexol (Omnipaque, 300 mg/mL iodine) with a power injector at a rate of 4 mL/second. When the arterial attenuation value (180–200 H) was reached following the injection of the contrasting agent, helical scanning was manually initiated. Around 150 to 300 axial images were obtained, and 2 to 10 reformatted images were constructed for the upper extremity in a CT 3D rendering workstation (Ultra 60, Sun Microsystems Inc, United States).

### Thrombectomy Procedure

In the first patient, a longitudinal medial incision was made at the right cubital fossa followed by dissection and isolation of the right brachial artery. Arteriotomy and subsequent thrombectomy using a 6-Fr Fogarty catheter (Edwards Lifesciences LLC, United States) was used to remove around 3- to 4-inch-long thrombus from the right brachial artery. To ensure adequate forward and backward blood flow following thrombectomy, 100,000 units of streptokinase and 500 units of heparin were infused. The incised artery was closed using Prolene 6/0 in a simple continuous fashion, and Nylon 3/0 was used for closing the skin. A similar procedure was used in the second patient to remove the thrombus from the right radial artery, and amputation of the entire thumb, index, and ring fingers, as well as the little finger at the middle phalanges level was performed. In the third patient, under general anesthesia, ultrasound-guided access to the right subclavian artery was achieved using a micropuncture technique. Subsequently, a thrombectomy was performed using a 3-Fr Fogarty catheter and the clot was removed.

### Enzymatic Assays

The venom metalloprotease activity was analyzed using DQ-gelatin (ThermoFisher Scientific, UK) as a fluorogenic substrate. Various concentrations of Russell's viper venom (Kentucky Reptile Zoo, United States) were mixed with 10 μg/mL DQ-gelatin in a total reaction volume of 100 μL (final volume was made up using PBS) in a 96-well microtiter plate. The plate was incubated at 37 °C, and the level of fluorescence was measured at various time points using an excitation wavelength of 485 nm and emission at 520 nm in a Fluostar Optima (BMG Labtech, Germany) spectrofluorometer. Similarly, the serine protease activity was measured using Nα-benzoyl- l -arginine 7-amido-4-methyl coumarin HCl (Sigma Aldrich, UK) as a fluorogenic substrate. Following the mixing of the substrate (2 μM) with different concentrations of Russell's viper venom, the plate was incubated at 37 °C and the resulting fluorescence was measured at an excitation wavelength of 366 nm and emission wavelength of 460 nm at various time points. The PLA _2_ activity of Russell's viper venom was measured using an EnzCheck Phospholipase A _2_ kit (ThermoFisher Scientific; dioleoyl phosphatidylcholine and dioleoyl phosphatidylglycerol as substrates) according to the manufacturer's instructions.

### Human Blood Collection and Preparation of Plasma/PRP

The blood samples from healthy human volunteers were collected in line with the procedures approved by the University of Reading Research Ethics Committee (UREC 17/17). Following written informed consent, blood was collected via venipuncture in vacutainers containing 3.2% (w/v) sodium citrate. To obtain PRP, blood was centrifuged at 100 g for 20 minutes at 20 °C in a benchtop centrifuge. The top layer of PRP was collected carefully without disturbing the white (opaque layer) or red blood cells and used in platelet aggregation assays. To obtain plasma, the whole blood was centrifuged at 1,400 *g* for 10 minutes at 20 °C. The top clear layer of plasma was carefully collected and used in clotting experiments. The citrated whole blood was used in ROTEM experiments without any modifications.

### Clotting Assays

PT and aPTT were measured using standard protocols in a Ceveron T100 fully automated coagulation analyzer (Technoclone, Austria). A concentration of 50 μg/mL of Russell's viper venom was mixed with a predetermined volume of plasma obtained from different donors and standard PT (thromboplastin and 25 mM CaCl _2_ )/aPTT (silica/sulfatide phospholipids in 25 mM CaCl _2_ ) reagents provided by the manufacturer and analyzed in Ceveron T 100.

### ROTEM Analysis

The ROTEM analysis was performed using a ROTEM Delta instrument (Werfen, UK) to determine the impacts of Russell's viper venom on various parameters of whole blood clotting. The Intem and Extem analyses were performed to determine the impact of venom on intrinsic and extrinsic (as well as common) pathways of blood clotting, respectively. Fibtem was performed to determine the impact of fibrinogen and clotting factors in whole blood clotting in the absence of platelets. Aptem was completed to analyze the impact of venom on blood clotting in the absence of fibrinolysis. For each assay, 50 μg/mL of Russell's viper venom was mixed with 300 μL of citrated human whole blood and pre-set volumes of respective reagents in line with the manufacturer's instructions. For Intem and Extem, the blood samples were recalcified using a Startem reagent (0.2 M CaCl _2_ in HEPES buffer, pH 7.4) and blood clotting was initiated using intrinsic (partial thromboplastin phospholipid from rabbit brain and ellagic acid) and extrinsic (recombinant tissue factor, phospholipids, and heparin) activators. For Fibtem, the blood was mixed with a Fibtem reagent (cytochalasin D and 0.2 M CaCl _2_ in HEPES buffer, pH 7.4) prior to the initiation of clotting using the Extem activator. For Aptem, the blood was mixed with Aptem reagent (aprotinin and 0.2 M CaCl _2_ in HEPES buffer, pH 7.4) prior to the activation of clotting using Extem activation reagent. To determine the impacts of marimastat and varespladib on Russell's viper venom-induced effects in Extem, 10 μM of each inhibitor was added with and without 50 μg/mL venom. The clot formation and lysis were monitored for 60 minutes in all assays.

### Flow Cytometry and Aggregation Assays

The PRP was incubated with various concentrations of Russell's viper venom for 5 minutes in the presence of 2 μg/mL FITC-labeled antihuman fibrinogen antibodies (Dako, UK) and 2 μg/mL PECy5-labeled CD62P (P-selectin) antibodies (BD Biosciences, UK). The platelets were then added either with modified Tyrode's HEPES buffer (2.9 mM KCl, 134 mM NaCl, 0.34 mM Na _2_ HPO _4_ .12H _2_ O, 1 mM MgCl _2_ , 12 mM NaHCO _3_ , 20 mM HEPES, pH 7.3) or 5 μM ADP (prepared in modified Tyrode's HEPES buffer), and further incubated for 5 or 20 minutes at 37 °C. Then 0.2% (v/v) formyl saline was used to fix the cells before analysis by flow cytometry (Accuri C6, BD Biosciences, UK). The median fluorescence intensity of each sample was collected by analyzing 5,000 cells within the gated region for platelets. The levels of median fluorescence intensity obtained with the relevant controls were taken as 100% to calculate the effects in venom-treated samples.

The aggregation assays were performed using an optical aggregometer (Chrono-Log, United States). The PRP was added with various concentrations of Russell's viper venom and incubated at 37 °C in an optimal aggregometer (Chrono-Log) while monitoring the level of aggregation for 5 minutes. Then ADP (5 μM) was added, and the baseline was set to 0, and the aggregation was monitored for another 5 minutes.

### In Vivo Thrombotic Assays in Mice

To assess whether Russell's viper venom induces intravascular thrombosis in vivo, experiments were performed in mice. Before injection, Russell's viper venom was incubated for 20 minutes at room temperature with a PLA _2_ inhibitor, varespladib (500 μM), to inhibit the neurotoxic PLA _2_ s of the venom. Then, a group of three CD-1 mice of both sexes (18–20 g) received an intravenous injection, in the caudal vein, of 12.5 μg venom, dissolved in a volume of 100 μL 0.12 M NaCl, 0.04 M phosphates, pH 7.2 (PBS). Another group of three control mice received 100 μL of PBS alone. Mice injected with varespladib-inhibited venom died within 15 minutes of injection, and control mice were sacrificed at the same time interval by an overdose of ketamine and xylazine. Immediately after death, the thoracic cavity of mice was opened, and tissue samples of lungs were collected and added to a formalin fixative solution. Tissues were processed for embedding in paraffin. Then, sections of 5 μm were obtained and stained with hematoxylin and eosin for histological observation. These experiments involving the use of mice were approved by the Institutional Committee for the Care and Use of Laboratory Animals (CICUA) of the University of Costa Rica (permission CICUA-025–15).

Similarly, to determine if Russell's viper venom can induce thrombosis in microvasculature and/or affected muscle tissues, the tibialis anterior muscle of mice (CD-1) was injected with Russell's viper venom (66 ng/g of mouse weight) and thereafter left to recover. The control mice were injected with an equal volume of PBS. Five days after damage, the mice were sacrificed and the muscle was processed for immunohistology analysis following cryosection (used 15-μm sections). The sections were incubated with FITC-labeled antifibrinogen antibodies (Dako, UK) and counterstained with DAPI to identify nuclei. These experiments involving the use of mice were approved by the Ethics Committee of the University of Reading and the British Home Office.

### Statistical Analysis

All data analyses in this study were performed using Prism 7 (GraphPad Inc, United States). The data are represented as mean ± SD. The statistical significance between control and treated samples was calculated using an ordinary one-way ANOVA with Dunnett's (or Turkey's test for ROTEM data with inhibitors) multiple comparisons test. Where only one concentration of venom was used to compare its effects with controls, an unpaired *t* -test was used.
